# Association between Alanine Aminotransferase/Aspartate Aminotransferase Ratio (AST/ALT Ratio) and Coronary Artery Injury in Children with Kawasaki Disease

**DOI:** 10.1155/2020/8743548

**Published:** 2020-03-23

**Authors:** Jinxin Wang, Jiawen Li, Yue Ren, Hongying Shi, Xing Rong, Xuting Zhang, Yiping Shao, Rongzhou Wu, Maoping Chu, Huixian Qiu

**Affiliations:** ^1^Children's Heart Center, The Second Affiliated Hospital and Yuying Children's Hospital, Institute of Cardiovascular Development and Translational Medicine, Wenzhou Medical University, Wenzhou 325027, Zhejiang, China; ^2^Department of Preventive Medicine, School of Public Health and Management, WenZhou Medical University, Wenzhou 325035, Zhejiang Province, China

## Abstract

**Objective:**

To investigate the association between the aspartate aminotransferase (AST)/alanine aminotransferase (ALT) ratio (AST/ALT ratio, AAR) and intravenous immunoglobulin (IVIG) resistance, coronary artery lesions (CAL), and coronary artery aneurysms (CAA) in children with Kawasaki disease (KD).

**Design:**

We retrospectively studied 2678 children with KD and divided them into two groups: a low-AAR group and a high-AAR group with a median AAR of 1.13 as the cut-off point. The differences in laboratory data, clinical manifestations, and coronary artery damage rates were compared between the two groups.

**Results:**

The incidence of CAL was higher in the low-AAR group than in the high-AAR group at 2 and 3-4 weeks after illness onset (*p* < 0.001, respectively). The IVIG resistance rate was significantly higher in the low-AAR group than in the high-AAR group (29.94% vs 21.71%, *p* < 0.001). The levels of C-reactive protein, erythrocyte sedimentation rate, white blood cell count, bilirubin, fibrinogen, thrombin time, D-dimer, and brain natriuretic peptide were also significantly higher in the low-AAR group compared with the high-AAR group. The levels of albumin and IgG were significantly lower in the low-AAR group compared with those of the high-AAR group. The proportion of typical KD cases in the low-AAR group was significantly higher than that in the high-AAR group. Low-AAR correlated with the risk of coronary artery damage and IVIG resistance.

**Conclusion:**

Children with KD who had low-AAR value were more likely to develop coronary artery damage and IVIG resistance. Low AAR is a risk factor for CAL, CAA, and IVIG resistance in KD.

## 1. Introduction

Kawasaki disease (KD) has been reported for more than 50 years [1], although its etiology is unknown. Its pathological features include extensive inflammation of small and medium blood vessels throughout the body. The major serious complication of KD is coronary artery lesions (CAL). Despite receiving intravenous immunoglobulin (IVIG) therapy timely, coronary artery aneurysms (CAAs) occur in 4% of patients with KD [2], and are the leading cause of acquired heart disease. Worldwide, the highest incidence per 100,000 (<5 year-old children) is in Japan (330) [3], followed by South Korea (194.7) [4], the USA (25) [5], the UK (8.39) [6], and Australia (9.34) [7]. Because of the lack of national statistics in China, only local data are available [8–12].

Transaminase has been used as a monitoring indicator of liver function for several decades and as a basic laboratory test in clinical practice [13]. Aspartate aminotransferase (AST) is widely found in the liver, kidney, brain, lung, and skeletal muscle, whereas alanine aminotransferase (ALT) is mainly present in the hepatocyte cytoplasm. When hepatocytes are damaged by acute inflammation, the cell membrane permeability increases, and AST and ALT are released into the blood although their concentrations and phases are different [14]. Clinically, AST/ALT ratio (AAR) is commonly used to test liver function. Abnormal conditions reflect damage to the liver cells. Elevated ALT is more representative of liver damage than AST because the elevation of AST is also caused by extrahepatic disease [15]. Early studies reported an association between AAR and chronic liver diseases such as immune liver disease, chronic viral hepatitis, and nonalcoholic liver disease [16–20]. Recently, AAR was reported to be associated with long-term cardiovascular disease [21]. Coronary damage in KD poses a hidden danger for late-onset cardiovascular events [22]. However, it is unclear whether there is any association between AAR and coronary artery damage in KD.

Therefore, we investigated whether AAR is associated with coronary artery damage in children with KD. To our knowledge, this is the first study to apply AAR in KD. The purpose of this study was to explore the association between AAR and coronary artery damage as well as IVIG resistance in children with KD.

## 2. Materials and Methods

### 2.1. Subjects

We reviewed the data of children with KD at the Second Affiliated Hospital of Wenzhou Medical University from 1 January 2011 to 31 December 2018. Overall, 2768 children were collected, of which 184 children without AST or ALT, 6 children with no cardiac ultrasound test, and 1 child with AST/ALT extreme outliers were excluded. Finally, 2577 children were included in the study. We divided all children with KD into two groups according to a median AAR value of 1.13: AAR < 1.13 = low-AAR group (1287 children) and AAR ≥ 1.13 = high-AAR group (1290 children).

All children were diagnosed with reference to the Japanese KD standard [23]. Children who met the 5 or 6/6 diagnostic criteria (fever >5 days; extremity changes; rash; conjunctivitis; oral changes; cervical lymphadenopathy) were defined as KD, and those with less than 5 diagnostic criteria were defined as atypical KD [24].

### 2.2. Data Collection and Laboratory Tests

We collected the following information: age (months), gender, time of IVIG treatment, whether receiving steroid therapy, and the method of IVIG treatment. We also collected laboratory data before IVIG treatment including white blood cell count, neutrophil count, hemoglobin level, lymphocyte count, eosinophil count, and levels of ALT, AST, C-reactive protein (CRP), erythrocyte sedimentation rate (ESR), and levels of albumin, sodium, potassium, fibrinogen, D-dimer, and the N-terminal prohormone of brain natriuretic peptide. Echocardiogram examinations were performed at 2 and 3–4 weeks after illness onset.

### 2.3. Resistance to IVIG

A body temperature >38.0°C that did not return to normal at 48 hours after the end of IVIG injection or an increase in body temperature >38.0°C within 2 weeks was defined as IVIG resistance [25].

### 2.4. CAL and CAA

CAL and CAA were defined as previously described [26]: (1) age <36 months, coronary diameter ≥2.5 mm; (2) 36 months ≥ age >108 months, coronary artery diameter ≥3 mm; and (3) age ≥108 months, a coronary artery diameter ≥3.5 mm was defined as a coronary lesion. A coronary artery diameter ≥4 mm was defined as a coronary artery aneurysm.

### 2.5. Statistical Analysis

SPSS version 23 was used for statistical analysis, and count data were expressed as the mean ± standard deviation or median and interquartile range, and measurement data were expressed as numbers and percentages. Categorical variables were compared using the Chi-square test. The relationship between AAR and coronary damage was tested using multiple logistic regressions. All tests were considered significant when *p* < 0.05.

## 3. Results

### 3.1. Demographic Characteristics and Clinical Data

As shown in Table 1, the proportion of male children was slightly higher than that in the high-AAR group compared with the low-AAR group. The time of IVIG treatment was earlier in the low-AAR group compared with the high-AAR group (6 days vs 7 days; *p* < 0.001). The proportion of IVIG no use was lower in the low-AAR group compared with the high-AAR group (3.43% vs 12.29%, *p* < 0.001). There were no differences in age and receiving steroid therapy before diagnosis between the high- and low-AAR groups. The proportions of principal symptoms such as rash, conjunctivitis, and changes of the extremities were higher in the low-AAR group compared with the high-AAR group (81.12% vs 70.16%, *p* < 0.001; 92.39% vs 80.70%, *p* < 0.001; 75.50% vs 69.77%, *p* = 0.001, respectively). As a result, the proportion of atypical KD was lower in the low-AAR group than in the high-AAR group (17.72% vs 31.55%, *p* < 0.001).

### 3.2. Laboratory Tests

As shown in Table 2, the levels of WBC and ANC were significantly higher in the low-AAR group than in the high-AAR group (15.83 × 10^9^/L vs 14.80 × 10^9^/L, *p* < 0.001; 10.50 × 10^9^/L vs 8.97 × 10^9^/L, *p* < 0.001). The levels of CRP (84.90 mg/L vs 60.45 mg/L, *p* < 0.001) and ESR (36.00 mm/h vs 34.00 mm/h, *p* < 0.001) were also higher in the low-AAR group compared with the high-AAR group. Platelet and lymphocyte counts were slightly lower in the low-AAR group compared with the high-AAR group (358 × 10^9^/L vs 373 × 10^9^/L, *p* < 0.001; 3.43 × 10^9^/L vs 3.63 × 10^9^/L, *p* < 0.001).

Regarding electrolyte measurements, the levels of sodium, potassium, chloride, calcium magnesium, and phosphate were slightly lower in the low-AAR group compared with the high-AAR group.

In the liver function test, except for the level of albumin in the low-AAR group, which was lower than in the high-AAR group, alanine aminotransferases, aspartate aminotransferase, glutamyl transpeptidase, and bilirubin were higher in the low-AAR group than in the high-AAR group. The levels of fibrinogen and D-dimers were slightly higher in the low-AAR group than in the high-AAR group (6.39 g/L vs 5.80 g/L, *p* < 0.001; 1.56 *μ*g/ml vs 1.20 *μ*g/ml, *p* < 0.001), and the level of thrombin time was slightly lower in the low-AAR group than in the high-AAR group (14.82 s vs 15.01 s, *p* < 0.001).

In addition, the level of NT-pro BNP was significantly higher in the low-AAR group than in the high-AAR group (1082.00 pg/mL vs 452.50 pg/ml, *p* < 0.001). The level of IgG was lower in the low-AAR group compared with the high-AAR group (694.00 mg/L vs 759.00 mg/L, *p* < 0.001). The proportions of patients with pyuria and proteinuria were higher in the low-AAR group than in the high-AAR group (44.19% vs 30.54%, *p* < 0.001; 41.32% vs 28.89%, *p* < 0.001).

### 3.3. Coronary Artery Outcomes and IVIG Resistance

As shown in Table 3, the proportion of IVIG resistance was significantly higher in the low-AAR group than in the high-AAR group (29.94% vs 21.71%, *p* < 0.001). Furthermore, the incidence of CAL was higher in the low-AAR group than in the high-AAR group at 2 weeks and 3-4 weeks after illness onset (29.46% vs 21.70%, *p* < 0.001; 24.11% vs 17.83%, *p* < 0.001). The incidence of CAA was also higher in the low-AAR group than in the high-AAR group at 2 weeks and 3-4 weeks after illness onset (3.63% vs 1.75%, *p*=0.004; 3.78% vs 1.52%, *p*=0.003).

### 3.4. Effect of AAR on CAL and IVIG Resistance

Logistic analysis was performed for the correlation between AAR and coronary artery damage and IVIG resistance (Table 4). The results showed that low-AAR correlated with a risk of IVIG resistance (OR = 1.542, 95% Cl = 1.279–1.858, *p* < 0.001). When we adjusted for possible confounding factors that might be associated with IVIG resistance, such as age, gender, IVIG treatment options, IVIG treatment time, value of AST, value of ALT, platelet count, neutrophil count, lymphocyte count, NT-pro BNP, immunoglobulin G, WBC count, albumin, and C-reactive protein levels, low-AAR remained a significant risk factor for IVIG resistance (OR = 1.452, 95% Cl = 1.036–2.034, *p*=0.0304). We also analyzed the correlation between AAR and coronary artery damage and found that low-AAR correlated with a risk of CAL at 2 and 3-4 weeks after illness onset (OR = 1.507, 95% Cl = 1.253–1.812, *p* < 0.001; OR = 1.464, 95% Cl = 1.168–1.836, *p*=0.001). Low-AAR also correlated with a risk of CAA at 2 and 3-4 weeks after illness onset (OR = 2.12, 95% Cl = 1.253–3.588, *p*=0.0051; OR = 2.545, 95% Cl = 1.360–4.763, *p*=0.0035). After adjusting for possible confounding factors, children with low-AAR were also more likely to develop CAL and CAA at 2 and 3-4 weeks after illness onset.

## 4. Discussion

A diagnosis of KD depends on subjective clinical manifestations and lacks objective gold criteria [27]. In recent years, the incidence of KD has gradually increased [4, 24], whereas the incidence of CAL has tended to be stable [27], resulting in an annual increase in the number of people with coronary artery lesions. Therefore, the early identification of risk factors for coronary artery damage in KD children is important.

KD occurs related to a wide range of small and medium blood vessel inflammation. In addition to the main damage to the coronary artery, the involvement of other blood vessels in the body was also reported [28]. The liver has a rich network of blood vessels including a hepatic artery and hepatic portal vein [29]. Therefore, liver function tests can be used to gain insights into liver changes in KD patients. The prediction of IVIG resistance has been evaluated by the following four IVIG resistance scoring systems [30–33]: the Egami score based on ALT, the Kobayshi score based on AST as a risk factor, San Diego based on glutamyl transpeptidase, and the Formosa score based on albumin. IVIG resistance was also reported as a risk factor for CAL [34].

In the present study, we found that ALT in the low-AAR group was significantly higher than in the high-AAR group (73.00 *μ*/L vs. 17.00 *μ*/L, *p* < 0.001) and that of AST was slightly higher (32.00 *μ*/L vs. 30.00 *μ*/L, *p* < 0.001). ALT is present in the cytoplasm of hepatocytes and its level increases; when the liver cell membrane is damaged, ALT will leak into the blood stream, leading to an increase in their plasma levels. AST is present mainly in the mitochondria of liver cells—not only in the liver, but also in the kidney, brain, lung, and skeletal muscle [15, 35]. Indeed, the half-life of ALT is shorter than for AST (17 and 47 h, respectively). Therefore, AST levels should undergo a more rapid change. But in KD, the changes in ALT levels were significantly greater than for AST, which may reflect the acute inflammatory response of the liver. In addition to ALT and AST, glutamyl transpeptidase and bilirubin were also higher, while the level of albumin was lower in the low-AAR group than in the high-AAR group.

AAR is commonly used to assess liver function and reflects the severity of liver disease. The first report on AAR appeared in 1957 [36], ten years before the first report of KD. Giannini et al. reported the utility of AAR in evaluating the prognosis of viral hepatitis [37]. Gurung et al. reported the association between AAR and the severity of alcoholic liver disease; increased AAR has been considered as an indicator that alcohol has induced mitochondrial damage, which causes a significant increase in AST [18]. AAR plays a role in detecting extrahepatic diseases, which is also used for assessing the prognosis of patients with tumor surgery. Huang et al. reported its use for the prognosis of patients with esophageal squamous cell carcinoma [38]. Lee et al. and Gorgel et al. reported the predictive effect of AAR in patients with urinary tract tumors after surgery [39, 40]. Therefore, AAR can be used for the diagnosis and prognosis of various diseases. In addition, the predictive utility of AAR was reported for acute ischemic stroke [41], cardiovascular events, [21] and peripheral arterial occlusive disease [42], where a group with AAR >1.67 had more frequent myocardial infarction, atrial fibrillation, and heart failure.

In the present study, our results also revealed that there were significant differences in the coronary artery damage between the two groups at 2 and 3–4 weeks after the onset of KD and IVIG resistance. Especially for the occurrence of CAA, the OR value of the low-AAR group was 2.6 times that of the high-AAR group, indicating that low AAR was a risk factor for CAL, CAA, and IVIG resistance. But these results are contrary to the predicted effects of the various reports mentioned above. For example, Zhao et al. [43] reported that high-AAR values were a risk factor for insulin resistance. Weng et al. [21] reported that in a 10-year long-term follow-up, a high-AAR value was a risk factor for men with cardiovascular disease. In addition, Canat et al. [44] reported that high-AAR values were risk factors for a poor prognosis of nonmetastatic renal cell carcinoma.

From the perspective of AAR reduction, we speculate that the change in the ratio reflects the inconsistency of ALT and AST. As mentioned above, ALT is mainly distributed in the liver cell cytoplasm, and AST is widely distributed in the muscle, brain, lung, kidney, and liver mitochondria [14]. The smaller the AAR value the more significant the ALT release is compared with AST. However, this does not mean that only ALT can be used to replace the AAR effect in the final logistic regression analysis, after adjusting for ALT and AST, the effects were consistent and more significant.

In the present study, AAR could be used to assess inflammation. The WBC, neutrophil count, levels of CRP, ESR, coagulation function index, liver enzyme, and BNP in the low-AAR group were higher than in the high-AAR group, whereas albumin, electrolytes, and IgG were lower than in the high-AAR group. In addition, we also observed slight differences in IVIG treatment options, but we believe that this is not the reason for the difference in the rate of coronary artery damage and IVIG resistance, because after adjusting for treatment type and treatment time, low-AAR remained a significant risk factor for CAL and IVIG resistance. For the clinical diagnosis and treatment, the low-AAR group received more formal treatment because of its more typical clinical manifestations, and the high-AAR group was not treated with IVIG or had delayed treatment because their clinical manifestations were not typical. However, in the low-AAR group, the standard and timely treatment had a worse prognosis, which strongly supports our conclusion. Therefore, AST/ALT ratio may be a good predictor that reflects the intensity of inflammation and predicts coronary artery damage in KD—the smaller the value of AAR, the stronger the acute inflammatory response, and the greater risk of coronary artery damage and IVIG resistance.

The limitations of this study included the loss of data regarding the height of KD children because of an update of the hospital's medical record system; thus, the Z value could not be used to calibrate the coronary damage results. However, we have reviewed the results of coronary arteries with Z values and the absolute diameter in recent years and found that the incidence of coronary artery damage and results did not change significantly. This partly explains why the absolute diameter of the coronary artery does not cause a significant bias in this study. In future research, we will use the Z value to further verify these findings. In addition, CRP [45], WBC [46], PLT [47], hemoglobin [48], albumin [49], and total bilirubin [50] have been reported as risk factors for coronary artery damage. Further studies are needed to investigate the superiority of low-AAR compared to the above mentioned risk factors. The strength of this study was the first use of AAR in KD using a large number of cases spanning more than 14 years. A larger and more in-depth study is needed to confirm our findings.

## 5. Conclusion

In our study, low-AAR was a risk factor for CAL, CAA, and IVIG resistance. AAR can effectively predict CAL, CAA, and IVIG resistance in KD. Therefore, in children diagnosed with KD admitted to hospital, clinicians should focus on AAR values and should not be misled by a simple abnormal value of the electronic system. In particular, children with low-AAR values should be monitored. Although the overall prognosis of KD is good, in the follow-up of low-AAR patients, especially at 3-4 weeks or later after the onset of the disease, it is necessary to carefully check echocardiograms and increase the frequency of follow-up.

## Figures and Tables

**Table 1:** Demographic characteristics.

	Low-AAR group	High-AAR group	*p* value
Number	1287	1290	
Age (month)	20.39 (11.85–35.77)	20.45 (9.99–38.83)	0.883
IVIG treat time (days)	6.00 (6.00–7.00)	7.00 (6.00–8.00)	<0.001
Sex			0.034
Male	821 (63.79%)	874 (67.75%)	
Female	466 (36.21%)	416 (32.25%)	
KD type			<0.001
Typical KD	1059 (82.28%)	883 (68.45%)	
Atypical KD	228 (17.72%)	407 (31.55%)	
Fever	1280 (99.46%)	1275 (98.84%)	0.088
Rash	1044 (81.12%)	905 (70.16%)	<0.001
Conjunctivitis	1189 (92.39%)	1041 (80.70%)	<0.001
Oral changes	1193 (92.70%)	1179 (91.40%)	0.222
Extremity changes	972 (75.52%)	900 (69.77%)	0.001
Lymph	635 (49.34%)	657 (50.93%)	0.419
IVIG treat			
No use	43 (3.43%)	156 (12.29%)	
2 g/kg^*∗*^day	1036 (82.62%)	974 (76.75%)	<0.001
1 g/kg^*∗*^day	175 (13.96%)	139 (10.95%)	
Steroid	80 (6.24%)	60 (4.66%)	0.079

Results are given as (*N*) mean (SD) median (Q1–Q3)/*N* (%). KD: Kawasaki disease; AAR: aspartate aminotransferase (AST)/alanine aminotransferase (ALT) ratio; IVIG: intravenous immunoglobulin therapy.

**Table 2:** Laboratory tests.

	Low-AAR group	High-AAR group	*p* value
Number (*n*)	1287	1290	
White blood cell count (10^9^/L)	15.83 (12.40–19.96)	14.80 (11.30–19.01)	<0.001
Hemoglobin (g/L)	110.16 (11.04)	110.70 (11.71)	0.227
Haematocrit (%)	0.33 (0.03)	0.33 (0.03)	0.170
Platelet count (10^9^/L)	358.00 (289.00–439.00)	373.00 (300.50–473.00)	<0.001
Neutrophil count (10^9^/L)	10.50 (7.74–14.21)	8.97 (6.25–12.30)	<0.001
Lymphocyte count (10^9^/L)	3.43 (2.24–4.89)	3.63 (2.44–5.28)	0.002
Eosinophil count (10^9^/L)	0.30 (0.12–0.57)	0.27 (0.10–0.55)	0.068
C-reactive protein (CRP, mg/L)	84.90 (49.89–132.00)	60.45 (27.95–101.25)	<0.001
Erythrocyte sedimentation rate (ESR, mm/h)	36.00 (27.00–45.00)	34.00 (24.00–43.00)	<0.001
Sodium (Na, mmol/L)	135.39 (2.66)	136.13 (2.55)	<0.001
Potassium (K, mmol/L)	4.25 (0.58)	4.41 (0.54)	<0.001
Chloride (Cl, mmol/L)	101.61 (3.34)	102.23 (3.06)	<0.001
Calcium (Ca, mmol/L)	2.27 (0.13)	2.33 (0.14)	<0.001
Magnesium (Mg, mmol/L)	0.91 (0.10)	0.93 (0.10)	<0.001
Phosphate (P, mmol/L)	1.37 (0.27)	1.48 (0.27)	<0.001
Alanine aminotransferase (ALT, *μ*/L)	73.00 (39.00–148.50)	17.00 (13.00–24.00)	<0.001
Aspartate aminotransferase (AST, *μ*/L)	34.00 (25.00–60.00)	30.00 (24.00–40.00)	<0.001
Albumin (g/L)	34.51 (6.42)	37.21 (6.24)	<0.001
Gamma-glutamyltransferase (*γ*-GT, *μ*/L)	89.00 (43.00–166.00)	16.00 (11.00–28.00)	<0.001
Total bilirubin (umol/L)	8.00 (5.20–13.20)	6.10 (3.90–8.90)	<0.001
Prothrombin time (PT, s)	13.99 (1.26)	13.64 (0.97)	<0.001
Activated partial thromboplastin time (APTT, s)	43.25 (6.61)	43.43 (6.27)	0.260
Fibrinogen (FIB, g/L)	6.39 (1.49)	5.80 (1.47)	<0.001
Thrombin time (s)	14.82 (1.01)	15.01 (1.29)	<0.001
D-dimers (*μ*g/ml)	1.56 (0.94–2.48)	1.20 (0.71–1.88)	<0.001
N-terminal prohormone of brain natriuretic peptide (NT-pro BNP, pg/ml)	1082.00 (464.50–2687.50)	452.50 (198.75–1052.50)	<0.001
Immunoglobulin G (IgG, mg/L)	694.00 (562.75–865.00)	759.00 (572.00–948.00)	<0.001
Immunoglobulin A (IgA, mg/L)	61.80 (42.18–96.53)	68.60 (42.10–111.75)	0.099
Immunoglobulin M (IgM, mg/L)	114.50 (84.57–151.00)	117.50 (82.40–156.00)	0.883
Urinary white cell (>5/HP)	373 (44.19%)	270 (30.54%)	<0.001
Urinary protein	350 (41.32%)	256 (28.89%)	<0.001
Urinary red cell (>3/HP)	90 (11.24%)	72 (8.52%)	0.065

Results are given as (*N*) Mean (SD) Median (Q1–Q3)/*N* (%).

**Table 3:** Study outcomes.

	Low-AAR group	High-AAR group	*p* value
IVIG-resistance	371 (29.94%)	244 (21.71%)	<0.001
CAL (2 weeks)	357 (29.46%)	261 (21.70%)	<0.001
CAL (3-4 weeks)	223 (24.11%)	164 (17.83%)	<0.001
CAA (2 weeks)	44 (3.63%)	21 (1.75%)	0.004
CAA (3-4 weeks)	35 (3.78%)	14 (1.52%)	0.003

CAL: coronary artery lesions; CAA: coronary artery aneurysm; AAR: aspartate aminotransferase (AST)/alanine aminotransferase (ALT) ratio; IVIG: intravenous immunoglobulin therapy.

**Table 4:** Multiple regression analysis.

	Nonadjusted	Adjusted
IVIG resistance		
Low-AAR group	1.0	1.0
High-AAR group	1.542 (1.279, 1.858) <0.0001	1.452 (1.036, 2.034) 0.0304
CAL (2 weeks)		
Low-AAR group	1.0	1.0
High-AAR group	1.507 (1.253, 1.812) <0.0001	1.956 (1.265, 3.024) 0.0025
CAL (3–4 weeks)		
Low-AAR group	1.0	1.0
High-AAR group	1.464 (1.168, 1.836) 0.0010	1.753 (1.125, 2.731) 0.0131
CAA (2 weeks)		
Low-AAR group	1.0	1.0
High-AAR group	2.120 (1.253, 3.588) 0.0051	2.751 (1.405, 5.386) 0.0032
CAA (3–4 weeks)		
Low-AAR group	1.0	1.0
High-AAR group	2.545 (1.360, 4.763) 0.0035	4.968 (1.061, 23.257) 0.0418

Statistic: *β* (95% CI) *p* value/OR (95% CI). IVIG resistance; CAL: coronary artery lesions; CAA: coronary artery aneurysm. Adjust model for age and gender; IVIG treatment options; IVIG treatment time; value of AST; value of ALT; platelet count; neutrophil count; lymphocyte count; NT-pro BNP; immunoglobulin G; white blood cell count; C-reactive protein; albumin level.

## Data Availability

The data used to support the findings of this study are available from the corresponding author upon request.
